# Green tea, other teas and coffee consumption and risk of death from chronic kidney disease as the underlying cause among Japanese men and women: the JACC Study

**DOI:** 10.1265/ehpm.24-00291

**Published:** 2025-03-05

**Authors:** Shuai Guo, Kazumasa Yamagishi, Tomomi Kihara, Isao Muraki, Akiko Tamakoshi, Hiroyasu Iso

**Affiliations:** 1 Doctoral Program in Public Health, Graduate School of Comprehensive Human Sciences, University of Tsukuba, Tsukuba, Japan; 2 Department of Public Health Medicine, Institute of Medicine, and Health Services Research and Development Center, University of Tsukuba, Tsukuba, Japan; 3 Department of Public Health, Graduate School of Medicine, Juntendo University, Tokyo, Japan; 4 Public Health, Department of Social Medicine, Osaka University Graduate School of Medicine, Suita, Japan; 5 Department of Public Health, Faculty of Medicine, Hokkaido University, Sapporo, Japan; 6 National Center for Global Health and Medicine, Bureau of International Cooperation, Institute for Global Health Policy Research (iGHP), Tokyo, Japan

**Keywords:** Green tea, Coffee, CKD, Mortality, Epidemiology

## Abstract

**Background:**

To explore the associations of green tea, coffee, black tea, and oolong tea consumption with mortality from chronic kidney disease (CKD) as the underlying cause among Japanese adults.

**Methods:**

We conducted a prospective cohort study of 110,585 men and women aged 40–79 years at recruitment from 1986 to 1990. Baseline information on the consumption of tea and coffee, lifestyles, and medical histories was obtained via self-administered questionnaires. We used multivariable Cox regression models to estimate sex-specific hazard ratios and 95% CIs of mortality from CKD associated with the consumption of green tea, coffee, black tea, or oolong tea.

**Results:**

After a median 19-year follow-up, the hazard ratios of mortality from CKD in women were 0.49 (95% CI, 0.22–1.06) for 1–2 cups of green tea per day, 0.56 (0.31–0.99) for 3–4 cups per day, and 0.55 (0.32–0.93) for ≥5 cups per day, compared with <1 cup per day. No such association was found in men. Coffee, black tea, and oolong tea consumption were not associated with CKD risk in either sex.

**Conclusions:**

Daily consumption of green tea was associated with a lower risk of mortality from CKD in women.

## 1. Introduction

Chronic kidney disease (CKD) is defined as a progressive and irreversible decline in renal function; it affects 9.1% of the population worldwide [1, 2]. Patient with CKD have increased risks of cardiovascular diseases, end stage renal disease (ESRD) and all-cause deaths, imposing a significant disease burden [3]. Although certain preventive measures, such as blood pressure and glucose control, have been developed, a practical and efficient method to delay the onset of CKD and CKD-associated adverse outcomes is still lacking.

In recent years, much scientific attention has been focused on the association between beverage intake and CKD. For example, several studies reported an inverse association between coffee consumption, one of the most commonly consumed beverages in Western societies, and risk of CKD [4–7]. This association is attributed to the effect of coffee increasing the antioxidant capacity and improving gut microbiome health and insulin sensitivity [8–10]. Green tea, which is commonly drunk in Asian societies, especially in Japan, is recognized as a powerful antioxidant and scavenger of reactive oxygen species [11]. Research related to these positive bioactivities has shown green tea to be inversely associated with the risk of diabetes [12], cardiovascular disease [13], and several cancers [14]. Notwithstanding, a previous prospective cohort study reported no association between green tea consumption and ESRD [6].

In the current study, we sought to determine whether consumption of green tea, coffee, black tea, or oolong tea is inversely associated with risk of CKD mortality as the underlying cause in the general Japanese population.

## 2. Participants and methods

### 2.1 Study population

**Fig. 1:**
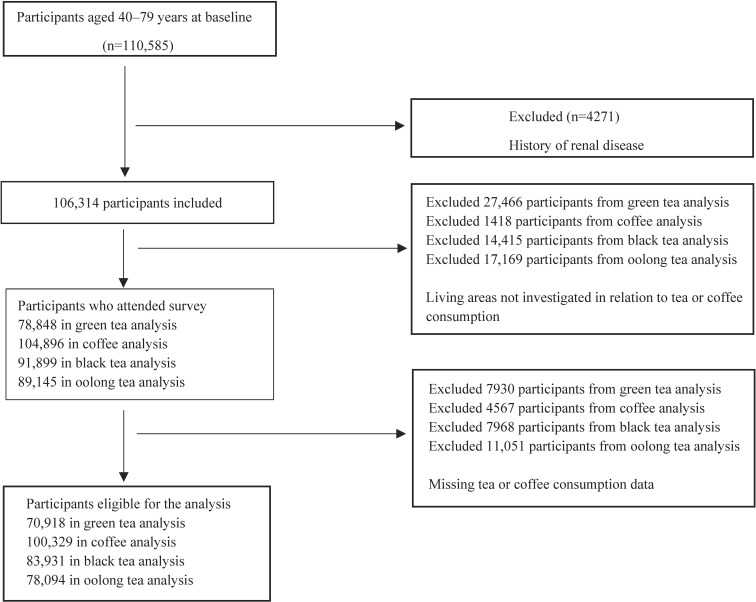
Flowchart of the participants selection process

The Japan Collaborative Cohort Study for Evaluation of Cancer Risk (JACC) Study is a large-scale population-based cohort study started between 1986 and 1990 [15]. A total of 110,585 individuals (46,395 men and 64,190 women) aged 40–79 years in 45 areas throughout Japan completed self-administered questionnaires regarding their lifestyles and medical histories. We excluded from the current analysis 4271 participants who had a previous history of renal disease. Then, we excluded 2 areas (n = 3321), 1 area (n = 1418), 7 areas (n = 14,415), 10 areas (n = 17,169) from our analyses of green tea, coffee, black tea, and oolong tea, respectively, because the baseline questionnaire did not include the items for green tea, coffee, black tea, or oolong tea. To explore the broader impact of green tea on CKD mortality, recognizing its prevalence as a daily beverage among Japanese individuals, we excluded 13 areas (n = 24,145) from our green tea analyses. These areas collected only data on the frequency of green tea consumption but did not survey the amount consumed. Furthermore, we excluded 7930 participants, 4567 participants, 7968 participants, and 11,051 participants from our analyses of green tea, coffee, black tea, and oolong tea, respectively, owing to missing data, In the end, 70,918 participants were included in our green tea analyses, 100,329 in our coffee analyses, 83,931 in our black tea analyses, and 78,094 in our oolong tea analyses (Fig. 1). Informed consent was obtained from the participants or local community leaders before the participants completed the questionnaires. The study was approved by the ethics committee of Hokkaido University.

### 2.2 Mortality surveillance

The median and maximum follow-up durations were 19 and 23 years, respectively. With the exception of 9 regions, the follow-up lasted until the end of 2009 (until 1999 in 4 areas, 2003 in 3 areas, and 2008 in 2 areas).

Mortality surveillance was filed in the public health center in the area of residency in each community and then sent centrally to the Ministry of Health and Welfare. The *International Classification of Diseases, Tenth Revision* (ICD-10) was used to code the underlying causes of death for the National Vital Statistics. Therefore, all deaths that occurred in the cohort were ascertained by use of death certificates from a public health center. If participants moved out of the communities or died of other causes, we treated them as censored participants.

We selected death from CKD as the endpoint (ICD10 codes E10.2–E10.29, E11.2–E11.29, E12.2, E13.2–E13.29, E14.2, I12–I13.9, N02–N08.8, N15.0, N18–N18.9) [16]. This definition includes not only CKD (N18–N18.9) but also CKD caused by diabetes (ICD10 codes E10.2–E10.29, E11.2–E11.29, E12.2, E13.2–E13.29, E14.2), CKD caused by hypertension (ICD10 codes I12–I13.9), and CKD caused by glomerulonephritis (ICD10 codes N03–N06.9).

### 2.3 Assessment of consumption of tea or coffee

Consumption of tea or coffee was assessed by use of a self-administered dietary questionnaire. Participants were questioned about their average consumption of green tea, black tea, oolong tea, or coffee during the previous year. The answer for frequency had 5 options: almost every day, 3–4 cups per week, 1–2 cups per week, 1–2 cups per month, and never. Participants who selected the response of “almost every day” were asked to state their average consumption of their beverage in numbers of cups per day. Given that over 80% of our participants reported daily green tea consumption, we combined the responses of “3–4 cups per week,” “1–2 cups per week,” “1–2 cups per month,” and “never” into a single category termed “<1 cup per day” and classified daily drinkers on the basis of their reported daily green tea consumption into 3 distinct groups: “1–2 cups per day,” “3–4 cups per day,” and “≥5 cups per day.” For coffee, we combined the “3–4 cups per week,” “1–2 cups per week,” “1–2 cups per month,” and “never” into “<1 cup per day” because of the small number of participants in these categories. Participants who selected “almost every day” were categorized as “≥1 cup per day”. We classified the categories of consumption of black tea or oolong tea as “<1 cup per week” and “≥1 cup per week” because black tea and oolong tea were not regularly consumed in this population. The common volume of a cup of tea or coffee was 100–120 ml.

The reproducibility of this dietary questionnaire has been described elsewhere [17]. The Spearman correlation coefficients between the 2 frequencies questionnaires, which were carried out at an interval of 1 year, were 0.62 for green tea, 0.86 for coffee, 0.74 for black tea, and 0.61 for oolong tea. Among men, the proportions consuming ≥1 cup per day were 83.7% for green tea, 33.5% for coffee, 2.4% for black tea, and 6.6% for oolong tea. In women, these proportions were 81.6%, 32.3%, 2.7%, and 10.7%, respectively.

### 2.4 Confounding factors

We adjusted for potential confounding factors including age; area of residence (Hokkaido, Tohoku, and Kanto; Chubu; Kinki; Chugoku and Kyushu); body mass index categories (<18.5, 18.5–25, and ≥25 kg/m^2^); smoking status (never, former, current smoker); ethanol intake (never, former, current drinker); exercising ≥5 h per week (yes or no); sleep duration (<7, 7–8, and ≥8 hours per day), history of hypertension and history of diabetes. All the items were collected through a self-administered questionnaire.

### 2.5 Statistical analysis

Descriptive data were expressed as means and standard deviations for the continuous variables and as numbers and percentages for the categorical variables. Tests for trends were conducted on the basis of a linear regression model or logistic regression model.

For each participant, person-years of follow-up were calculated from the date of completion of the baseline questionnaire to death, relocation, or the end of follow-up, whichever occurred first. The hazard ratio based on the Cox proportional hazard model for mortality from CKD was calculated for each category of beverage consumption. For each beverage consumption, the lowest consumption was used as the reference category. We estimated the age- and area-adjusted hazard ratios and the multivariable hazard ratios in the Cox regression models, stratified by sex. Missing values for the confounding factors were treated as additional missing categories and their dummy variables were included in the model.

Because renal function decline is associated with death from any cause [3], deaths due to other causes may preclude CKD death. Therefore, we also examined the association of green tea or coffee consumption with CKD mortality using competing risks analysis with the Fine and Gray model. Deaths from all other causes were considered competing risk events. Considering that the presence of cancer or cardiovascular disease can lead to lifestyle changes, we also conducted the analysis excluding participants with a history of cancer, stroke, or myocardial infarction.

All the analyses were performed with SAS version 9.4 (SAS Institute, Inc.), and significance was based on a 2-tailed probability of 0.05.

## 3. Results

Table 1 shows the participants’ baseline characteristics according to consumption of green tea, coffee, black tea, or oolong tea. Higher consumption of green tea was positively correlated with age in both sexes. Furthermore, those with higher green tea consumption exhibited the lowest prevalence of diabetes, the lowest percentage of participants who drank alcohol, and the highest percentage of history of hypertension and participants engaging in exercise ≥5 h per week in men and women. When compared with the participants who consumed <1 cup of coffee per day, those who consumed ≥1 cups per day were younger and had a shorter sleep duration, a lower prevalence of hypertension and diabetes, a higher percentage of smoking, and a lower percentage of engaging in exercise ≥5 h per week in men and women, a higher percentage of current alcohol consumption in women, and a lower percentage of current alcohol consumption in men. Similar differences were found between participants who consumed <1 cup of black tea per week and those who consumed ≥1 more cups per week, except in sleep duration, smoking status, and exercise. Compared with participants who consumed <1 cup of oolong tea per week, those who consumed ≥1 cups per week were younger and had a shorter sleep duration, a higher prevalence of diabetes in men and women, and a higher percentage of current drinking in women.

**Table 1:** Baseline characteristics according to consumption of tea and coffee, black tea, and oolong tea

**Variable**	**Participants, n**	**Age, y^a^**	**Body mass index, ** **kg/m^2 a^**	**Sleep duration (hours ** **per day)^a^**	**History of hypertension, ** **n (%)**	**History of diabetes, ** **n (%)**	**Current smoker, ** **n (%)**	**Current drinker, ** **n (%)**	**Sports participation ≥5 h/week, ** **n (%)**
Men									
Green tea									
<1 cup/day	4708	56.1 ± 10.4	22.7 ± 2.8	7.5 ± 1.2	834 (17.7)	323 (6.9)	2330 (49.5)	3398 (72.2)	314 (6.8)
1–2 cups/day	4599	55.9 ± 10.6	22.6 ± 2.8	7.4 ± 1.1	830 (18.1)	270 (5.9)	2422 (52.7)	3396 (73.8)	314 (6.8)
3–4 cups/day	7236	57.4 ± 10.4	22.6 ± 2.8	7.4 ± 1.1	1336 (18.5)	493 (6.8)	3619 (50.0)	5343 (73.8)	489 (6.8)
≥5 cups/day	13,329	58.4 ± 9.8	22.6 ± 2.8	7.5 ± 1.1	2554 (19.2)	771 (5.8)	6919 (51.9)	9425 (70.7)	1020 (7.7)
P for trend		<0.001	0.29	0.21	0.05	0.009	0.02	0.01	<0.001

Coffee									
<1 cup/day	28,041	58.9 ± 9.9	22.6 ± 2.8	7.6 ± 1.2	5962 (21.3)	1858 (6.6)	12,825 (45.7)	20,495 (73.1)	1745 (6.2)
≥1 cup/day	14,138	54.8 ± 10.2	22.7 ± 2.8	7.3 ± 1.0	2011 (14.2)	717 (5.1)	8667 (61.3)	10,028 (70.9)	807 (5.7)
P for trend		<0.001	0.99	<0.001	<0.001	<0.001	<0.001	<0.001	<0.001

Black tea									
<1 cup/week	30,916	57.3 ± 10.1	22.6 ± 2.8	7.5 ± 1.1	5941 (19.2)	1875 (6.1)	16,012 (51.8)	22,495 (72.8)	2095 (6.8)
≥1 cup/week	4219	57.0 ± 10.7	22.7 ± 2.8	7.4 ± 1.1	704 (16.7)	232 (5.5)	1972 (46.7)	2957 (70.1)	288 (6.8)
P for trend		0.14	0.13	0.79	<0.001	0.25	<0.001	0.01	0.81

Oolong tea									
<1 cup/week	27,361	57.5 ± 10.2	22.5 ± 2.7	7.5 ± 1.1	5180 (18.9)	1544 (5.6)	14,091 (51.5)	19,806 (72.4)	1862 (6.8)
≥1 cup/week	5472	55.2 ± 9.9	23.3 ± 2.9	7.4 ± 1.6	1041 (19.0)	418 (7.6)	2774 (50.7)	3934 (71.9)	404 (7.4)
P for trend		<0.001	<0.001	0.01	0.86	<0.001	0.65	0.48	0.16

Women									
Green tea									
<1 cup/day	7610	56.5 ± 10.1	22.9 ± 3.2	7.1 ± 1.1	1492 (19.6)	324 (4.3)	392 (5.2)	1787 (23.5)	289 (3.8)
1–2 cups/day	5385	56.2 ± 10.4	22.7 ± 3.1	7.0 ± 1.1	987 (18.3)	181 (3.4)	292 (5.4)	1335 (24.8)	225 (4.2)
3–4 cups/day	10,843	58.0 ± 10.3	22.7 ± 3.0	7.1 ± 1.1	2219 (20.5)	400 (3.7)	432 (4.0)	2356 (21.7)	458 (4.2)
≥5 cups/day	17,208	58.4 ± 9.7	22.9 ± 3.1	7.1 ± 1.1	3570 (20.8)	592 (3.4)	766 (4.5)	3553 (20.7)	811 (4.7)
P for trend		<0.001	<0.001	0.78	0.04	0.002	<0.001	<0.001	<0.001

Coffee									
<1 cup/day	39,350	59.3 ± 9.8	23.0 ± 3.2	7.2 ± 1.3	9073 (23.1)	1605 (4.1)	1319 (3.4)	7490 (19.0)	1549 (3.9)
≥1 cup/day	18,800	54.5 ± 9.8	22.8 ± 3.0	6.9 ± 1.1	2918 (15.5)	476 (2.5)	1454 (7.7)	5539 (29.5)	657 (3.5)
P for trend		<0.001	<0.001	<0.001	<0.001	<0.001	<0.001	<0.001	<0.001

Black tea									
<1 cup/week	41,698	57.6 ± 10.0	22.9 ± 3.1	7.1 ± 1.1	8791 (21.1)	1548 (3.7)	1900 (4.6)	8657 (20.8)	1729 (4.2)
≥1 cup/week	7098	56.7 ± 10.4	22.6 ± 3.0	7.0 ± 1.1	1314 (18.5)	206 (2.9)	278 (3.9)	1809 (25.5)	315 (4.4)
P for trend		0.26	<0.001	0.59	<0.001	0.003	0.04	<0.001	0.26

Oolong tea									
<1 cup/week	35,679	57.8 ± 10.1	22.7 ± 3.1	7.1 ± 1.1	7354 (20.6)	1223 (3.4)	1487 (4.2)	7185 (20.1)	1544 (4.3)
≥1 cup/week	9582	55.8 ± 9.6	23.5 ± 3.1	7.0 ± 1.0	2072 (21.6)	406 (4.2)	594 (6.2)	2588 (27.0)	378 (3.9)
P for trend		<0.001	<0.001	0.009	0.06	<0.001	<0.001	<0.001	0.09

^a^Mean ± standard deviation

As shown in Table 2, consumption of green tea was inversely associated with risk of mortality from CKD only in women. With reference to participants who consumed <1 cup of green tea per day, the multivariable-adjusted hazard ratios were 0.49 (95% CI, 0.22–1.06) in those who consumed 1–2 cups of green tea per day, 0.56 (0.31–0.99) in those who consumed 3–4 cups per day, and 0.55 (0.32–0.93) in those who consumed ≥5 cups per day. On the other hand, we did not observe any significant associations of coffee, black tea, or oolong tea consumption with CKD mortality in either men or women.

**Table 2:** Association of tea and coffee consumption with mortality from chronic kidney disease

**Variable**	**Men**				**Women**			
	
	**Number at risk, n**	**Death of CKD, n**	**Age and area-adjusted HR** **(95% CI)**	**Multivariable HR** **(95% CI)^a^**	**Number at risk, n**	**Death of CKD, n**	**Age and area-adjusted HR** **(95% CI)**	**Multivariable HR** **(95% CI)^a^**
Green tea								
<1 cup/day	4708	14	1	1	7610	24	1	1
1–2 cups/day	4599	11	0.80 (0.36–1.77)	0.83 (0.37–1.84)	5385	9	0.48 (0.22–1.05)	0.49 (0.22–1.06)
3–4 cups/day	7236	26	1.10 (0.57–2.12)	1.12 (0.58–2.17)	10,843	24	0.54 (0.30–0.96)	0.56 (0.31–0.99)
≥5 cups/day	13,329	65	1.22 (0.67–2.22)	1.20 (0.66–2.19)	17,208	44	0.55 (0.33–0.93)	0.55 (0.32–0.93)
Increase per cup	-	-	1.04 (0.99–1.10)	1.04 (0.99–1.10)	-	-	0.95 (0.89–1.02)	0.95 (0.89–1.02)

Coffee								
<1 cup/day	28,041	140	1	1	39,350	142	1	1
≥1 cup/day	14,138	37	0.82 (0.56–1.19)	0.85 (0.58–1.23)	18,800	25	0.71 (0.46–1.10)	0.78 (0.50–1.22)
Increase per cup	-	-	0.91 (0.74–1.13)	0.92 (0.74–1.14)	-	-	0.73 (0.52–1.05)	0.80 (0.57–1.14)

Black tea								
<1 cup/week	30,916	118	1	1	41,698	113	1	1
≥1 cup/week	4219	15	0.92 (0.53–1.57)	0.95 (0.55–1.63)	7098	14	0.82 (0.47–1.43)	0.87 (0.50–1.53)
Increase per cup	-	-	0.61 (0.17–2.16)	0.64 (0.18–2.22)	-	-	1.08 (0.59–1.97)	1.05 (0.58–1.89)

Oolong tea								
<1 cup/week	27,361	99	1	1	35,679	91	1	1
≥1 cup/week	5472	24	1.41 (0.90–2.21)	1.25 (0.79–1.96)	9582	23	1.20 (0.75–1.91)	1.03 (0.64–1.66)
Increase per cup	-	-	1.05 (0.89–1.25)	1.03 (0.86–1.23)	-	-	1.05 (0.90–1.23)	1.02 (0.87–1.19)

Abbreviations: CKD, chronic kidney disease; HR, hazard ratio; CI, confidence interval.

^a^Further adjusted for body mass index, smoking status, drinking status, exercise habits, sleep duration, history of hypertension and history of diabetes.

In our competing risks analysis, the results were unchanged, and the association between those who consumed ≥1 cups of green tea per day and CKD mortality in women remained consistent: The hazard ratios of mortality from CKD were 0.50 (95% CI, 0.23–1.10), 0.57 (0.32–1.01), and 0.59 (0.36–0.98) in women who consumed 1–2, 3–4, and ≥5 cups of green tea per day, respectively.

When we excluded participants with a history of cancer, stroke, or myocardial infarction at baseline, the results remained unchanged (data not shown), although the association between consuming 3–4 cups of green tea per day and CKD mortality in women became insignificant due to limited statistical power. As compared with those who consumed <1 cup of green tea per day, the multivariable-adjusted hazard ratios for women were 0.53 (95% CI, 0.24–1.15) for those who consumed 1–2 cups per day, 0.59 (0.32–1.07) for those who consumed 3–4 cups per day, and 0.57 (0.33–0.98) for those who consumed ≥5 cups per day. For men, the hazard ratios were 0.90 (0.39–2.11), 1.19 (0.59–2.42), and 1.35 (0.71–2.56), respectively.

The results of the association between coffee consumption and CKD mortality remained unchanged in both men and women after exclusion of those had a history of cancer, stroke, or myocardial infarction at baseline, and in the competing risk analysis.

## 4. Discussion

We found an inverse association between green tea consumption and risk of CKD mortality in women but not in men. Compared with women who consumed <1 cup of green tea per day, those who consumed ≥1 cups of green tea per day had a lower risk of CKD mortality. We did not observe any association between consumption of green tea and CKD mortality in men or between consumption of coffee, black tea, or oolong tea and CKD mortality in either men or women.

Tea, originating from China, is generally categorized into 3 major types according to the manufacturing process: green tea (non-fermented), oolong tea (partially fermented), and black tea (fermented). Owing to the higher content of antioxidant compounds in green tea than in black tea and oolong tea [18–20], green tea has been receiving much attention in terms of its possible preventive effects and use as a treatment for several diseases such as multiple cancers, metabolic syndrome, and ESRD associated with oxidative stress [21–24].

Epidemiologic studies focusing on the association between tea consumption and CKD have been scarce and inconsistent. However, both animal and human clinical studies have indicated that supplementation of catechin has significant protective effects against deterioration of renal function [21, 22, 25–27]. A Mendelian randomization study using data from a UK biobank reported an inverse association between tea intake and risk of CKD and albuminuria and a positive association between tea intake and estimated glomerular filtration rate (eGFR) [28]. In 2 other prospective cohort studies respectively conducted in Iran and the Netherlands, tea consumption was not associated with CKD nor with an annual change in eGFR [29, 30]. Regretfully, neither of these studies classified the type of tea. A prospective cohort study conducted in Singapore, of 63,257 Chinese adults aged 45–74 years, found no association between either green or black tea consumption and risk of ESRD [6]. Few studies have been conducted on this topic so far, and the current study is the first to show an inverse association between green tea consumption and CKD mortality.

We speculated that this association is related mainly to the antioxidative activity of green tea. Previous research has demonstrated the oxidative stress involved in all stages of CKD, ranging from the early stages to ESRD [31, 32]. The catechins present in green tea are widely recognized for their potent antioxidant property [11]. In addition to the antioxidative activity, anti-inflammatory activity, and antihypertensive effects of green tea, catechin could contribute to a reduced risk of CKD [33]. Our study showed different results between men and women. We speculate that these discrepancies are attributable to the influence of sex hormones. A previous double-blind randomized clinical trial suggested that the administration of green tea extract significantly increased circulating estradiol concentrations in healthy postmenopausal women [34]. Estrogen may exert protective effects on the progression of CKD by reducing oxidative stress [35]; inducing the release of nitric oxide synthesis [36]; reducing total cholesterol, low-density lipoprotein, and triglyceride levels [37]; and increasing high-density lipoprotein levels [38].

In addition to green tea, coffee consumption is on the rise among the Japanese population. However, contrary to previous studies [4–6], our study did not find a significant association between coffee consumption and risk of CKD mortality. The aforementioned study conducted in Singapore reported an inverse association between coffee consumption and risk of ESRD: Compared with those who consumed no coffee to less than 1 cup weekly, the multivariable hazard ratios were 0.91 (95% CI, 0.79–1.05) for those who consumed 1 cup of coffee per day (one-third of the total population) and 0.82 (0.71–0.96) for those who consumed ≥2 cups per day (another one-third of the total population) [6]. In our study, the proportions of those who drank 1 cup of coffee per day and of those who drank ≥2 cups per day were only 15% and 19%, respectively. Because the number of participants who drank coffee daily was limited in our study, we may not have detected an association between coffee consumption and risk of CKD mortality. Besides, coffee consumption is frequently associated with unhealthy behaviors, such as smoking and physical inactivity. In our study, those who consumed ≥1 cups of coffee per day accounted for the higher proportion of participants who smoked and the lower proportion of those engaging in exercise ≥5 hours per week. Although we adjusted for these participants, the unmeasured residual confounding probably weakened the association between coffee consumption and risk of CKD mortality in our study.

Our study has several limitations. First, we used only the dietary information collected at baseline, whereas the frequency of coffee and tea consumption may vary with time. However, as outlined in the Methods section, the repeatability of the consumption of tea and coffee, which was compared by 2 administrations of the food frequency questionnaire over a 1-year period, exhibited moderate-to-high correlations [17]. According to the National Health and Nutrition Survey in Japan, per capita consumption of tea was 310 g in 2002 and 293 g in 2011, indicating a relatively stable trend, whilst the corresponding consumptions of coffee and cocoa were 65 g and 123 g, suggesting that coffee consumption patterns may have changed in recent years. Besides, it is possible that catechin intake from green tea has declined in recent years due to the increased consumption of bottled green tea. A previous study demonstrated that autoclaving green tea for 20 minutes at 120 °C resulted in approximately a 20% reduction in catechin content, suggesting that industrial processing methods may diminish total catechin levels [39]. Second, since the assessment of these beverages relied on self-administered questionnaires, the misclassification of consumption status was inevitable. In our previous study, the Spearman correlation coefficient between a food frequency questionnaire of green tea consumption and a weighed record method for 12 days was 0.21 [17]. Such undifferential misclassification in cohort studies could introduce bias, potentially leaning results toward the null hypothesis. Third, although we excluded a subgroup of patients on the basis of a self-reported history of renal disease, we did not have data on serum creatinine levels or proteinuria to assess renal function at baseline. Therefore, some undiagnosed or preclinical, but not overt, CKD might be included in the population at baseline. Fourth, the use of CKD mortality based on the underlying cause as an outcome may lead to undercoding of early-stage CKD, thus failing to capture the full spectrum of the disease [40]. In addition, in the present study, CKD patients who died from other causes, such as cardiovascular disease, were not classified as having CKD as the underlying cause of death. This may have led to an underestimation of the numbers of CKD-associated mortalities, potential to influence our results. On the other hand, risk factors for them are thought to be common with those for cardiovascular disease. Thus, our outcome setting attempted to explore more specific risk factors for CKD. Finally, some exposure categories have a limited number of CKD deaths, yielding low statistical power.

In conclusion, our cohort study showed that daily consumption of green tea was associated with a lower risk of CKD mortality in women but not in men.

## Abbreviations

CKD: chronic kidney disease
ESRD: end-stage renal disease
eGFR: estimated glomerular filtration rate
JACC study: The Japan Collaborative Cohort Study for Evaluation of Cancer Risk Study
ICD-10: The International Classification of Diseases, Tenth Revision

## Declarations

### Data availability statement

The data underlying this article will be shared on reasonable request to the corresponding author.
